# Morphology, genetic characterization and molecular phylogeny of pinworm *Skrjabinema longicaudatum* n. sp. (Oxyurida: Oxyuridae) from the endangered Tibetan antelope *Pantholops hodgsonii* (Abel) (Artiodactyla: Bovidae)

**DOI:** 10.1186/s13071-020-04430-6

**Published:** 2020-11-11

**Authors:** Yi-Fan Cao, Hui-Xia Chen, Yang Li, Dang-Wei Zhou, Shi-Long Chen, Liang Li

**Affiliations:** 1 Key Laboratory of Adaptation and Evolution of Plateau Biota (AEPB), Northwest Institute of Plateau Biology, Chinese Academy of Sciences, Xining, 810008 Qinghai People’s Republic of China; 2 Qinghai Key Laboratory of Animal Ecological Genomics, Northwest Institute of Plateau Biology, Chinese Academy of Sciences, Xining, 810008 People’s Republic of China; 3 Key Laboratory of Animal Physiology, Biochemistry and Molecular Biology of Hebei Province, College of Life Sciences, Hebei Normal University, Shijiazhuang, 050024 Hebei Province People’s Republic of China; 4 University of Chinese Academy of Sciences, Beijing, 100093 People’s Republic of China

**Keywords:** Tibetan antelope, Parasite, Nematoda, Morphology, Genetic data, Phylogeny

## Abstract

**Background:**

The Tibetan antelope *Pantholops hodgsonii* (Abel) (Artiodactyla: Bovidae) is an endangered species of mammal endemic to the Qinghai-Tibetan Plateau. Parasites and parasitic diseases are considered to be important threats in the conservation of the Tibetan antelope. However, our present knowledge of the composition of the parasites of the Tibetan antelope remains limited.

**Methods:**

Large numbers of nematode parasites were collected from a dead Tibetan antelope. The morphology of these nematode specimens was observed using light and scanning electron microscopy. The nuclear and mitochondrial DNA sequences, i.e. small subunit ribosomal DNA (*18S*), large subunit ribosomal DNA (*28S*), internal transcribed spacer (ITS) and cytochrome *c* oxidase subunit 1 (*cox*1), were amplified and sequenced for molecular identification. Moreover, phylogenetic analyses were performed using maximum likelihood (ML) inference based on *28S* and *18S* + *28S* + *cox*1 sequence data, respectively, in order to clarify the systematic status of these nematodes.

**Results:**

Integrated morphological and genetic evidence reveals these nematode specimens to be a new species of pinworm *Skrjabinema longicaudatum* (Oxyurida: Oxyuridae). There was no intraspecific nucleotide variation between different individuals of *S. longicaudatum* n. sp. in the partial *18S*, *28S*, ITS and *cox*1 sequences. However, a high level of nucleotide divergence was revealed between the new species and its congeners in *28S* (8.36%) and ITS (20.3–23.7%) regions, respectively. Molecular phylogenetic results suggest that the genus *Skrjabinema* should belong to the subfamily Oxyurinae (Oxyuroidea: Oxyuridae), instead of the subfamily Syphaciidae or Skrjabinemiinae in the traditional classification, as it formed a sister relationship to the genus *Oxyuris*.

**Conclusions:**

A new species of pinworm *Skrjabinema longicaudatum* n. sp. (Oxyurida: Oxyuridae) is described. *Skrjabinema longicaudatum* n. sp. represents the first species of Oxyurida (pinworm) and the fourth nematode species reported from the Tibetan antelope. Our results contribute to the knowledge of the species diversity of parasites from the Tibetan antelope, and clarify the systematic position of the genus *Skrjabinema*. 
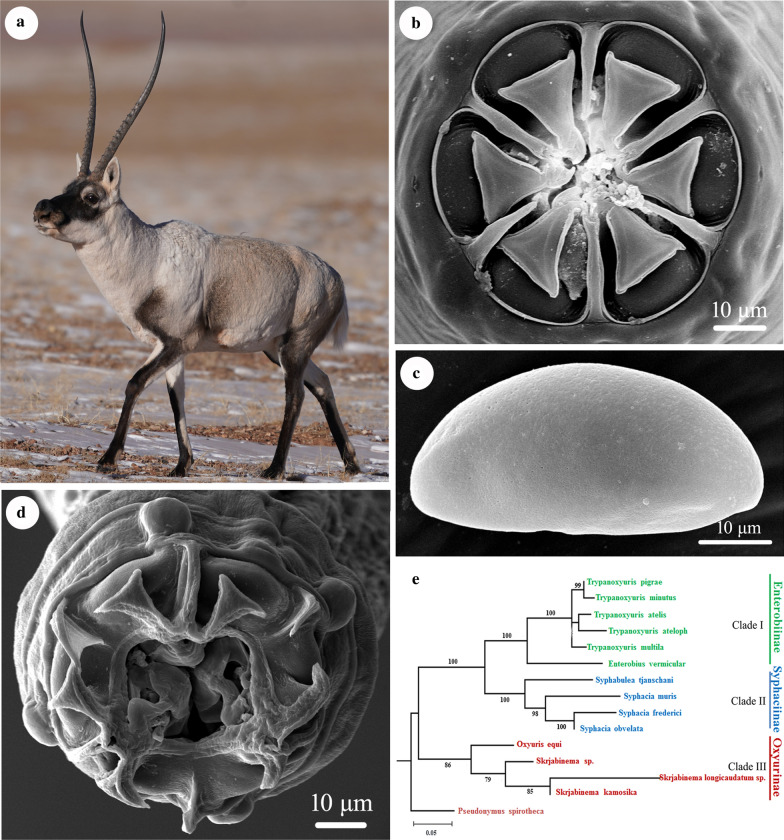

## Background

The Tibetan antelope *Pantholops hodgsonii* (Abel) (Artiodactyla: Bovidae) is an endangered species of mammal endemic to the Qinghai-Tibetan Plateau. The population of the Tibetan antelope has been declined severely, because of the loss and fragmentation of its habitat and commercial poaching [1, 2]. The latest estimate of the global population of the Tibetan antelope is 100,000–150,000 mature individuals (https://www.iucnredlist.org/species/15967/50192544). This species is listed as “Near Threatened” in the IUCN Red List of Threatened Species™ and also listed as Class I (Endangered in China) National Protected Wild Animal Species in China.

Parasites and parasitic diseases are considered to be important threats in wildlife conservation, as they can potentially impair the health of wildlife, decrease fitness, cause population declines and even contribute to local extinction [3–7]. Parasites are also significant pathogens of the Tibetan antelope [1, 2]. To date, 17 species of ectoparasites and endoparasites have been reported from the Tibetan antelope, including 5 species of oestrid and hippoboscid flies, 7 species of protozoans, 2 species of tapeworms and 3 species of nematodes [1, 2, 8–12].

In the present study, some nematode specimens were collected from the digestive tract of the Tibetan antelope, which were identified morphologically as a new species of the genus *Skrjabinema* (Oxyurida: Oxyuridae) using light and scanning electron microscopy. The nuclear and mitochondrial DNA sequences, i.e. small subunit ribosomal DNA (*18S*), large subunit ribosomal DNA (*28S*), internal transcribed spacer (ITS) and cytochrome *c* oxidase subunit 1 (*cox*1), were also amplified and sequenced for molecular identification of this species. Moreover, in order to clarify the systematic status of the genus *Skrjabinema*, phylogenetic analyses were performed using maximum likelihood (ML) inference based on *28S* and *18S* + *28S* + *cox*1 sequence data, respectively.

## Methods

### Parasite collection

A Tibetan antelope died naturally in the Hohxil National Nature Reserve, Qinghai Province, China. The digestive tract of this Tibetan antelope was sent to the Key Laboratory of Adaptation and Evolution of Plateau Biota (AEPB), Northwest Institute of Plateau Biology, Chinese Academy of Sciences for examination of parasites. Large numbers of nematode parasites were isolated from the caecum and colon. Specimens were fixed and stored in in 5% glycerine plus 70% ethanol until study.

### Morphological observations

For light microscopical studies, nematodes were cleared in lactophenol. Drawings were made using a Nikon microscope drawing attachment. For scanning electron microscopy (SEM), the anterior and posterior parts of specimens were re-fixed in 4% formaldehyde solution, post-fixed in 1% OsO4, dehydrated *via* an ethanol series and acetone, and then critical-point dried. Samples were coated with gold and examined using a Hitachi S-4800 scanning electron microscope at an accelerating voltage of 20 kV. Measurements (the range, followed by the mean values in parentheses) are given in micrometers (μm) unless otherwise stated.

### Molecular protocols

Three female specimens were randomly chosen for molecular analysis. Genomic DNA from each sample was extracted using a Column Genomic DNA Isolation Kit (Shanghai Sangon, China) according to the manufacturer’s instructions. The partial *18S* region was amplified by polymerase chain reaction (PCR) using the forward primer 18S-F (5'-CGC GAA TRG CTC ATT ACA ACA GC-3') and the reverse primer 18S-R (5'-GGG CGG TAT CTG ATC GCC-3') [13]. The partial *28S* region was amplified by PCR using the forward primer 28S-F (5'-AGC GGA GGA AAA GAA ACT AA-3') and the reverse primer 28S-R (5'-ATC CGT GTT TCA AGA CGG G-3') [14]. The ITS-1 region of nuclear rDNA was amplified by PCR using the forward primer SS1 (5'-GTT TCC GTA GGT GAA CCT GCG-3') and the reverse primer SS2R (5'-AGT GCT CAA TGT GTC TGC AA-3'). The ITS-2 region of nuclear rDNA was amplified by PCR using the forward primer NC13 (5'-ATC GAT GAA GAA CGC AGC-3') and the reverse primer NC2 (reverse: 5'-TTA GTT TCT TTT CCT CCG CT-3') [15]. The partial *cox*1 region was amplified by PCR using the forward primer *cox*1F2020 (5'-GAG TAC TAA TCA TAA GGA TAT TGG-3') and the reverse primer *cox*1R2020 (5'-ACA TAA ACY TCA GGA TGA CCA-3'), both newly designed in present study. The cycling conditions are as described previously [16]. PCR products were checked on GoldView-stained 1.5% agarose gels and purified with Column PCR Product Purification Kit (Shanghai Sangon, China). Sequencing was carried out using a Dye Deoxy Terminator Cycle Sequencing Kit (v.2, Applied Biosystems, California, USA) and an automated sequencer (ABI-PRISM 377). Sequences were aligned using ClustalW2. The DNA sequences obtained herein were compared (using the algorithm BLASTn) with those available in the National Center for Biotechnology Information (NCBI) database (https://www.ncbi.nlm.nih.gov).

### Phylogenetic analyses

Phylogenetic trees were constructed using maximum likelihood (ML) inference with MEGA X software based on the partial *28S* and *18S *+ *28S *+ *cox*1 sequence data, respectively. *Pseudonymus spirotheca* (Oxyurida: Thelastomatoidea: Pseudonymidae) was treated as the outgroup. The ingroup includes the representatives of the Oxyuridae with the *28S* and *18S *+ *28S *+ *cox*1 sequence data available in the GenBank database. We used a built-in function in the software MEGA X to select a best-fitting substitution model for the present sequences according to the Bayesian information criterion. The K2 (Kimura 2-parameter) + G model for the *28S* sequence data, and the HKY (Hasegawa-Kishino-Yano) + G + I model for the *18S*+*28S*+*cox*1 sequence data were identified as the optimal nucleotide substitution model, respectively. Nodal support for ML trees were tested using 1000 bootstrap replications, and bootstrap values exceeding 80% were showed in the phylogenetic trees.

## Results

Family Oxyuridae Cobbold, 1864

Genus *Skrjabinema* Werestschagin, 1926

### *Skrjabinema longicaudatum* n. sp.

#### Type-host

Tibetan antelope *Pantholops hodgsonii* (Abel) (Artiodactyla: Bovidae: Caprinae).

#### Type-locality

Hoh Xil Nature Reserve near Wudaoliang (35° 26′ N, 93° 17′ E), Qinghai Province, China.

#### Type-specimens

Holotype: male (HBNU-N-2020M001L); allotype: female (HBNU-N-2020M002L); paratypes: 9 females (HBNU-N-2020M003L) deposited in the College of Life Sciences, Hebei Normal University, Hebei Province, China; paratypes: 2 males and 100 females (KLAEPB No.019001) deposited in the Key Laboratory of Adaptation and Evolution of Plateau Biota, Northwest Institute of Plateau Biology, Chinese Academy of Sciences, Qinghai Province, China.

#### Site in host

Caecum and colon.

#### Prevalence and intensity

A single Tibetan antelope examined with 124 worms.

#### ZooBank registration

To comply with the regulations set out in Article 8.5 of the amended 2012 version of the *International Code of Zoological Nomenclature* (ICZN) [17], details of the new species have been submitted to ZooBank. The Life Science Identifier (LSID) of the article is urn:lsid:zoobank.org:pub:9194626F-7C3B-445C-BD36-0AF06E39C46F. The LSID for the new name *Skrjabinema longicaudatum* is urn:lsid:zoobank.org:act:3A5AB2D4-5B82-4CBE-8CF8-76B97783694E.

#### Etymology

The specific epithet is derived from a combination of the Latin words *longus*- (long) and *caudatum*- (cauda), and refers to the unusually long tail in the female of the new species.

## Description

### General

**Fig. 1:**
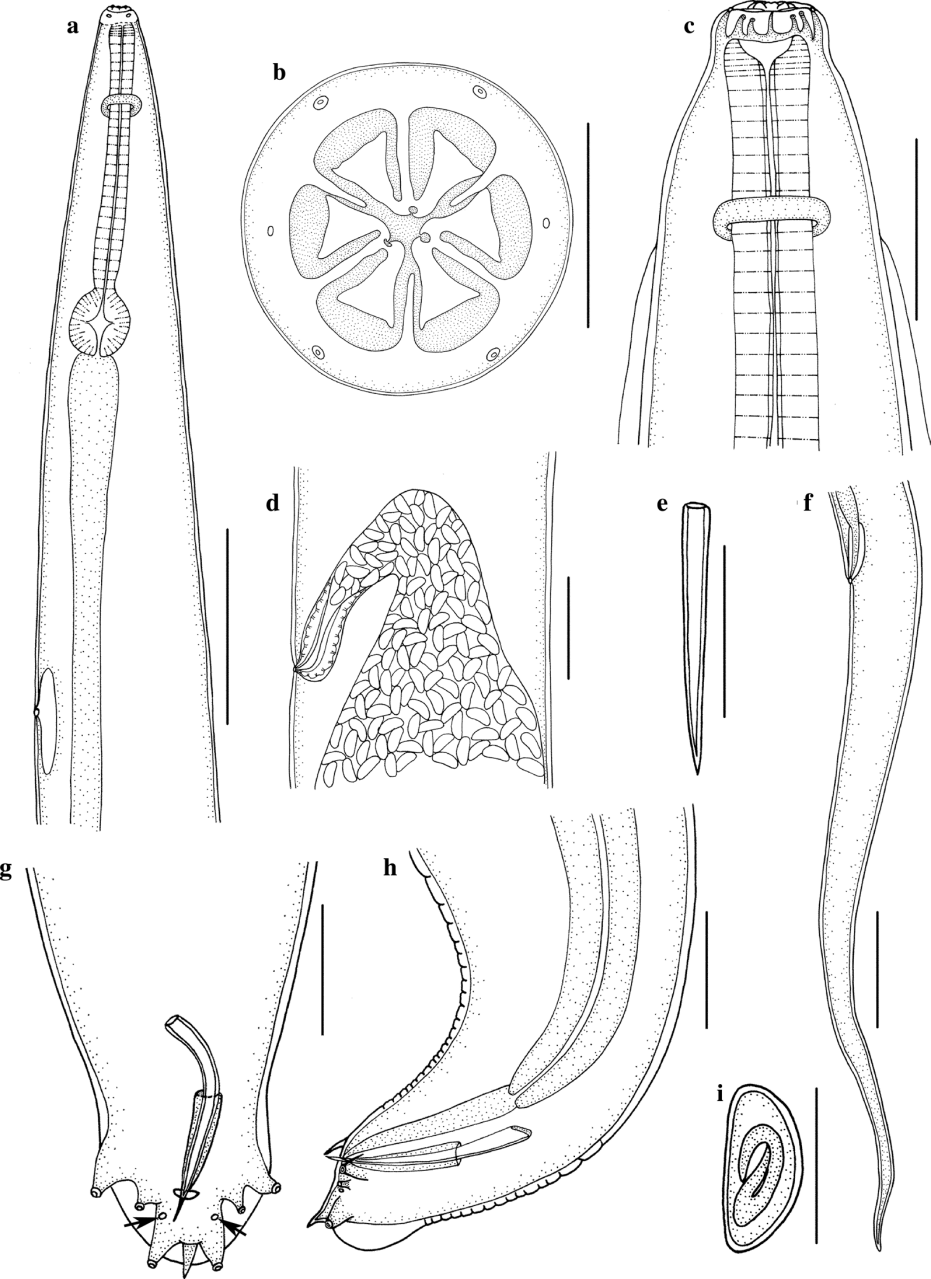
*Skrjabinema longicaudatum* n. sp. (Oxyurida: Oxyuridae) from the endangered Tibetan antelope *Pantholops hodgsonii* (Abel) (Artiodactyla: Bovidae) in China. **a** Anterior part of female, lateral view. **b** Cephalic extremity of female, apical view. **c** Anterior part of male, dorsal view. **d** Region of vulva, lateral view. **e** Spicule. **f** Posterior extremity of female, lateral view. **g** Posterior extremity of male (phasmids arrowed), ventral view. **h** Posterior extremity of male, lateral view. **i** Egg. *Scale-bars*: **a**, **f**, 500 μm; **b**, **e**, **g**, **h**, **i**, 50 μm; **c**, 100 μm; **d**, 200 μm

**Fig. 2:**
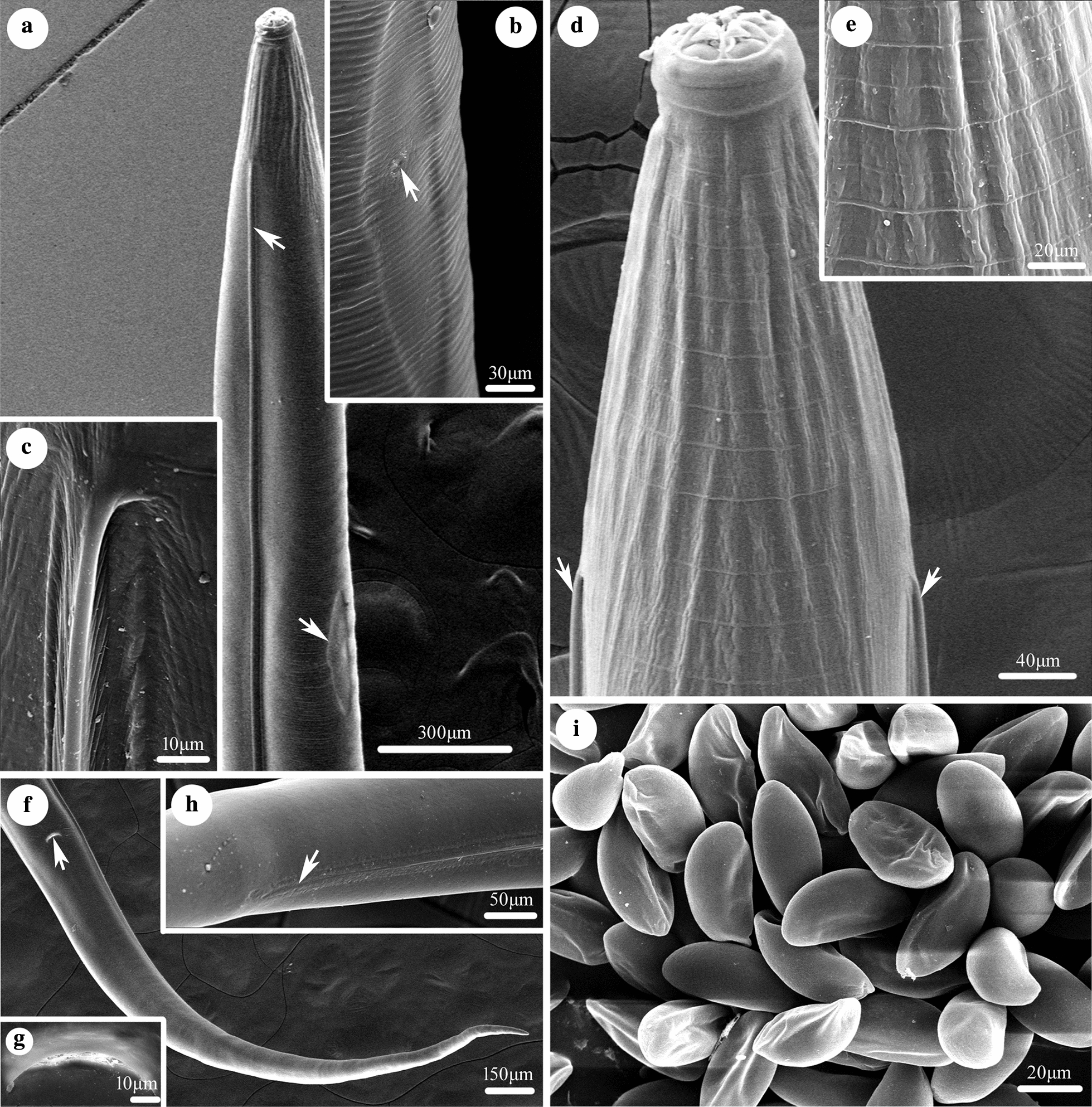
Scanning electron micrographs of *Skrjabinema longicaudatum* n. sp. (Oxyurida: Oxyuridae) from the endangered Tibetan antelope *Pantholops hodgsonii* (Abel) (Artiodactyla: Bovidae) in China. **a** Anterior part of female (lateral ala and depressed region around excretory pore arrowed), lateral view. **b** Magnified image of depressed region and excretory pore (excretory pore arrowed). **c** Magnified image of the original position of lateral ala. **d** Anterior part of female (lateral alae arrowed), dorsal view. **e** Magnified image of transverse annulations in the anterior part of body. **f** Posterior extremity of female (anus arrowed), ventral view. **g** Magnified image of anus. **h** Magnified image of the ending position of caudal ala. **i** Magnified image of eggs in uterus in different views

**Fig. 3:**
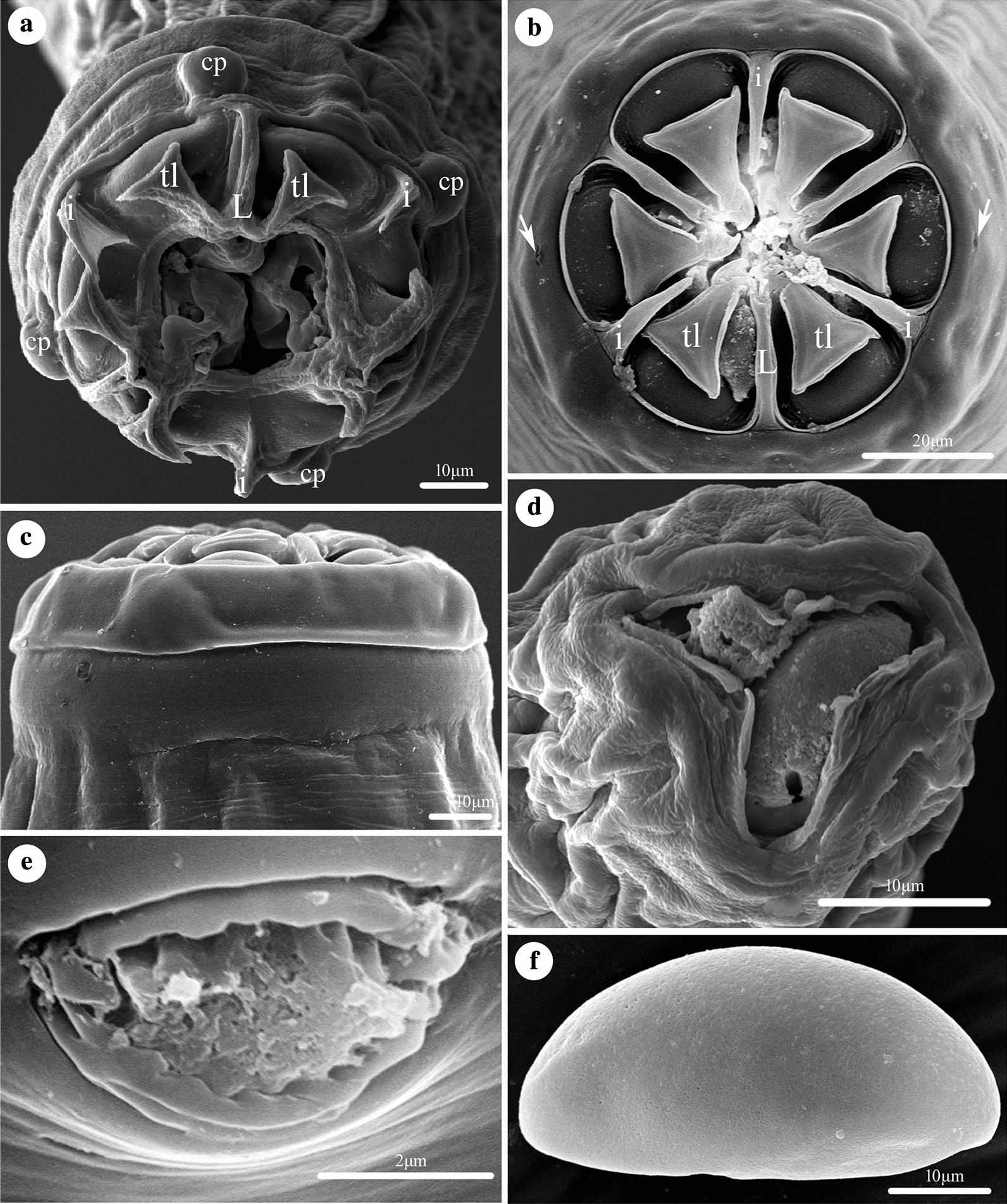
Scanning electron micrographs of *Skrjabinema longicaudatum* n. sp. (Oxyurida: Oxyuridae) from the endangered Tibetan antelope *Pantholops hodgsonii* (Abel) (Artiodactyla: Bovidae) in China. **a** Cephalic region of female, sub-apical view. **b** Cephalic region of female (amphidial pores arrowed), apical view. **c** Cephalic region of female, lateral view. **d** Cephalic region of male, apical view. **e** Magnified image of vulva. **f** Magnified image of egg, lateral view

Small-sized, whitish nematodes. Body cylindrical, maximum width at slightly posterior to mid-body. Cephalic vesicle indistinct in both sexes (Fig. 1a, c). Lateral alae present in both sexes (Figs. 1c, 2a, c, d). Sexual dimorphism prominent in cephalic structure (Figs. 1b, 3a, b, d). Cuticle with remarkable transverse annulations in anterior part of body (Fig. 2d, e). Buccal cavity very small, without cuticular tooth or other ornamentation. Oesophagus divided into short pharynx, cylindrical corpus, indistinct isthmus and ovoid posterior bulb with valves (Fig. 1a). Nerve-ring situated at about 1/4 of total oesophageal length (Fig. 1a). Excretory pore located in body wall depression, posterior to oesophago-intestinal junction (Figs. 1a, 2a, b). Deirids not observed.

### Male

[Based on 3 mature specimens; Figs. 1c, e, g, h, 3d]: Body 1.92–2.85 (2.38) mm long; maximum width 141–151 (146). Oral aperture simple, triradiate, surrounded by three small, more or less triangular lips with small apical median notch (Fig. 3d). Interlabia or interlabial projections absent (Fig. 3d). Oesophagus 400–454 (427) in total length, representing 15.7–20.9 (17.6)% of body length; pharynx + corpus + isthmus 255‒308 (280) long, size of bulb 143–146 (145) × 131–132 (132). Nerve-ring at 170–200 (185) and excretory pore at 760–890 (828) from cephalic extremity, respectively. Lateral alae narrow, extending from about level of nerve-ring to anterior region of cloaca (Fig. 1c). Posterior extremity of body distinctly curved ventrally (Fig. 1h). Spicule single, pointed at distal end, 74–81 (76.7) long, representing 3.15–3.85 (3.43) % of body length (Fig. 1e, g, h). Gubernaculum small, well sclerotized, about 48 long (Fig. 1g, h). Caudal papillae large, 3 pairs in total, arranged as follows: 1 pair precloacal, 1 pair paracloacal and 1 pair postcloacal (Fig. 1g). Preventral, median finger-like protuberance present (Fig. 1h). Tail 33 long, ending in short finger-like tip (Fig. 1g, h). Phasmids present slightly posterior to cloaca.

### Female

[Based on 10 mature specimens; Figs. 1a, b, d, f, i, 2a–i, 3a–c, e, f): Body 9.92‒12.1 (11.1) mm long; maximum width 396‒574 (475). Cephalic extremity with three anchor-shaped lips, each lip with 2 triangular lateral lobes not attached to cephalic rim (Figs. 1b, 3a–c). Interlabia digitiform, between lateral lobes of lips (Figs. 1b, 3a, b). Four large cephalic papillae and 2 small amphidial pores present (Figs. 1b, 3a, b). Oesophagus 832‒881 (853) in total length, representing 6.98‒8.78 (7.75) % of body length; pharynx + corpus + isthmus 634‒703 (671) long, size of bulb 168‒188 (181) × 139‒188 (161). Nerve-ring at 198‒248 (225) and excretory pore at 1.24‒1.92 (1.77) mm from cephalic extremity, respectively. Lateral alae extending from long distance posterior to base of cephalic extremity and ending at about middle of tail (Fig. 2a, c, d, f, h). Vulva a transverse slit, very small, with rudimentary lips observed under SEM, located at 2.97‒3.37 (3.16) mm from cephalic extremity, representing 25.1‒31.1% (28.7%) of body length (Figs. 1d, 3e). Egg asymmetrical, flattened at one side, embryonated or nonembryonated, thick-shelled, with smooth surface, 40‒59 (51) × 20‒40 (30) (*n* = 20) (Figs. 1i, 2i, 3f). Anus with small pre anal lip (Fig. 2g). Tail slender, very long, 2.63‒3.13 (2.90) mm, with pointed tip, representing 23.0‒27.7 (26.3) % of body length (Figs. 1f, 3f). Phasmids not observed.

## Genetic characterization

### Partial 18S region

Three *18S* sequences of *S. longicaudatum* n. sp. obtained herein were all 678 bp in length and represent one genotype. There are two species of *Skrjabinema* with *18S* sequence registered in GenBank, namely *S. kamosika* (AB699690) and *Skrjabinema* sp. (EF180060). Pairwise comparison of *18S* sequences between *S. longicaudatum* n. sp. and the two species of *Skrjabinema* displayed 0.29‒1.18% nucleotide divergence. The *18S* sequences of *S. longicaudatum* n. sp. are deposited in the GenBank database under the accession numbers MW020179-MW020181.

### Partial ITS region

Three ITS sequences of *S. longicaudatum* n. sp. obtained herein were all 1079 bp in length and represent one genotype. There are two species of *Skrjabinema* with ITS sequence registered in GenBank, namely *S. kamosika* (AB699691) and *Skrjabinema* sp. (AB367796). Pairwise comparison of ITS sequences between *S. longicaudatum* n. sp. and the other two species of *Skrjabinema* displayed 20.3‒23.7% nucleotide divergence. The ITS sequences of *S. longicaudatum* n. sp. are deposited in the GenBank database under the accession numbers MW020057-MW020059.

### Partial 28S region

Three *28S* sequences of *S. longicaudatum* n. sp. obtained herein were all 819 bp in length and represent one genotype. There is only one species of *Skrjabinema*, namely *S. ovis* (KY990019) with *28S* sequence registered in GenBank. Pairwise comparison of ITS sequences between *S. longicaudatum* n. sp. and *S. ovis* displayed 8.36% nucleotide divergence. The *28S* sequences of *S. longicaudatum* n. sp. are deposited in the GenBank database under the accession numbers MW020098-MW020100.

### Partial cox1 region

Three *cox*1 sequences of *S. longicaudatum* n. sp. obtained herein was 360 bp in length. There is no species of *Skrjabinema* with *cox*1 sequence registered on GenBank. The *cox*1 sequences of *S. longicaudatum* n. sp. are deposited in the GenBank database under the accession numbers MW021552-MW021554.

### Phylogenetic analyses

**Fig. 4:**
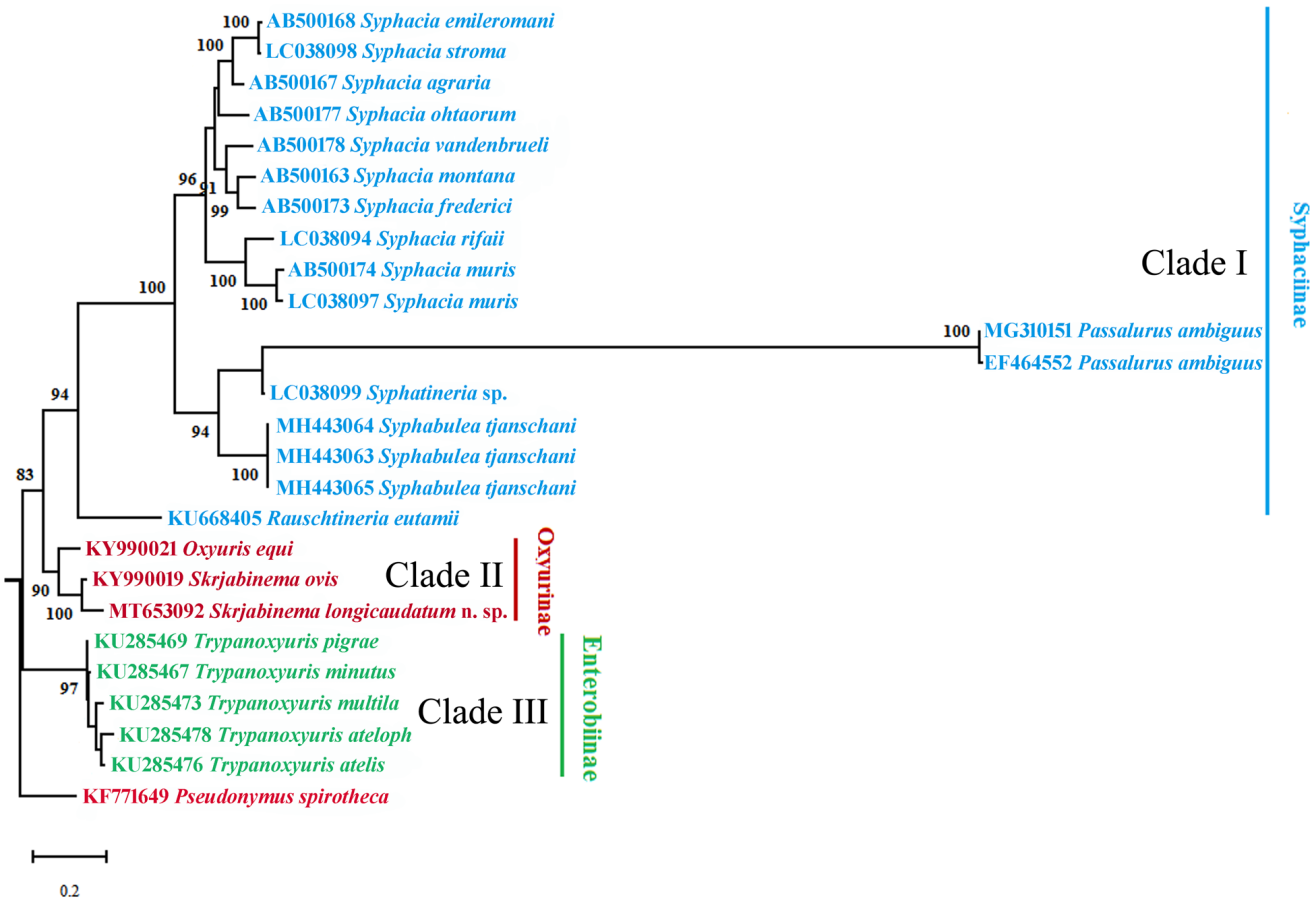
Maximum likelihood (ML) tree constructed from the partial 28S gene data showing the phylogenetic relationships of representatives of the family Oxyuridae. *Pseudonymus spirotheca* (Oxyurida: Pseudonymidae) was chosen as the outgroup

Phylogenetic trees based on the partial *28S* sequence data showed that representatives of the family Oxyuridae were divided into three monophyletic clades. Clade I included members of the genera *Syphacia*, *Passalurus*, *Syphatineria*, *Syphabulea* and *Rauschtineria*, representing the subfamily Syphaciinae. Clade II contained species of the genera *Oxyuris* and *Skrjabinema*, representing the subfamily Oxyurinae. Clade III included species of the genus *Trypanoxyuris*, representing the subfamily Enterobiinae (Fig. 4). *Skrjabinema longicaudatum* n. sp. displayed a sister relationship to *S. ovis*.

**Fig. 5:**
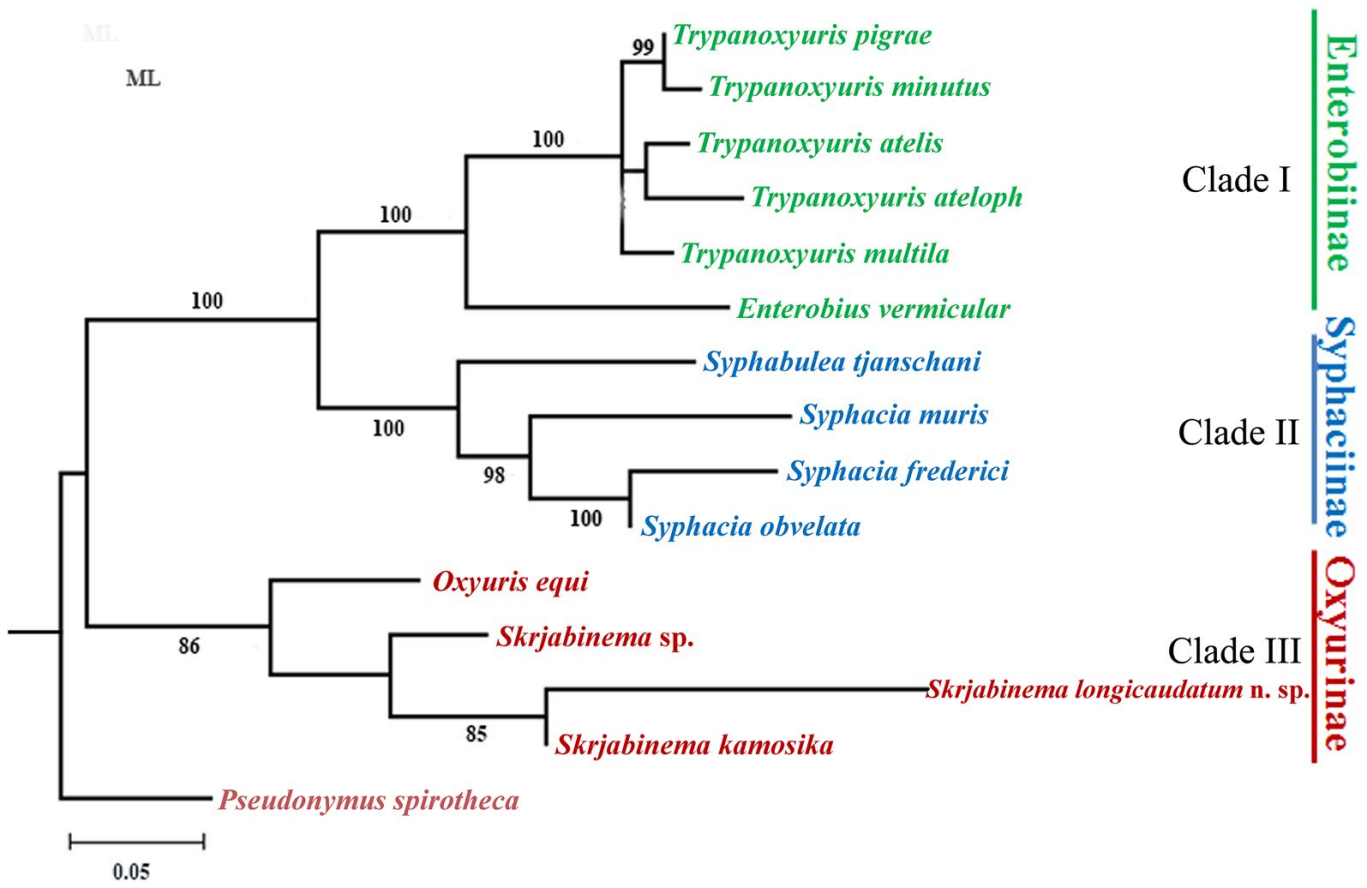
Maximum likelihood (ML) tree constructed from the partial 18+28S+*cox*1 gene data showing the phylogenetic relationships of representatives of the family Oxyuridae. *Pseudonymus spirotheca* (Oxyurida: Pseudonymidae) was chosen as the outgroup

Phylogenetic tree constructed based on the *18S*+*28S*+*cox*1 sequence data had similar topology to the phylogenetic results using the partial *28S* sequence data, in which representatives of the Oxyuridae also divided into three monophyletic clades (Fig. 5). Species of *Trypanoxyuris* and *Enterobius* formed clade I, representing the subfamily Enterobiinae. The members of *Syphabulea* and *Syphacia* grouped together (clade II), belonging the subfamily Enterobiinae. Clade III included representatives of *Oxyuris* and *Skrjabinema*, representing the subfamily Oxyurinae. *Skrjabinema longicaudatum* n. sp. clustered together with *S. kamosika*.

## Discussion

The genus *Skrjabinema* Werestschagin, 1926 (Oxyuridea: Oxyuridea) currently includes 10 nominal species reported from various ruminants worldwide, namely *S. alata* Mönnig, 1932, *S. africana* Mönnig, 1932, *S. caprae* Schad, 1959, *S. chubuki* Gagarin & Sapozhnikov, 1968, *S. kamosika* Hasegawa, Sato, Suzuki & Kaneshiro, 2012, *S. ovis* (Skrjabin, 1915), *S. parva* Dikmans, 1942, *S. rupicaprae* Böhm & Gebauer, 1930, *S. skrjabini* Gagarin & Sapozhnikov, 1968 and *S. tarandi* Skrjabin & Mizkewitsch, 1930 [18–24]. However, some of these species have not been sufficiently well described, especially the details of cephalic structure.

*Skrjabinema ovis* is the type-species of this genus, which has been widely reported from goats and sheep in Asia, Europe, America and Australia [21]. This species has been recorded from *Capra aegagrus hircus* (Linnaeus), *Ovis aries* Linnaeus and *Procapra przewalskii* Büchner in China [25]. The new species differs from *S. ovis* in the absence of cephalic vesicle in females (*vs* the presence of remarkable cephalic vesicle in the female in *S. ovis*), slightly shorter spicules (0.074–0.081 mm long in the new species *vs* 0.09–0.12 mm long in *S. ovis*) and longer gubernaculum (0.048 mm long in *S. longicaudatum* n. sp. *vs* 0.019‒0.025 mm long in the latter). *Skrjabinema longicaudatum* n. sp. has the spicule without a dilated proximal end, longer gubernaculum (0.019‒0.025 mm long) and the caudal alae ending about half-way along the tail in female, which is different from that of *S. parva* (the proximal end of spicule extends into a goblet-shaped, the gubernaculum 0.01‒0.016 mm long and the caudal alae ending close to the tail tip in female). The absence of sub-interlabial projections in the cephalic region in the male distinguishes the new species from *S. kamosika*, *S. tarandi* and *S. caprae* (the presence of sub-interlabial projections in the cephalic end in the male). Moreover, the caudal alae of the female in *S. tarandi* and *S. caprae* are very long (ending near the tail tip *vs* caudal alae ending at about half the tail length in the female of *S. longicaudatum*). *Skrjabinema longicaudatum* n. sp. differs from *S. rupicaprae* by having a larger body size of male (1.92–2.85 *vs* 1.54–1.79 mm long in *S. rupicaprae*), a relatively shorter spicule (spicule representing 3.15–3.85 % of body length in the former *vs* representing 4.47–5.20 % of body length in the latter) and a slightly longer gubernaculum (0.048 mm long in the new species *vs* 0.025 mm long in *S. rupicaprae*). The new species can be distinguished from *S. chubuki* and *S. skrjabini* by having morphologically different lips in the female (triangular lateral lobes of lip small, not attached to the cephalic rim in *S. longicaudatum* n. sp. *vs* triangular lateral lobes of lip very large, attached to the cephalic rim in the latter two species).

Mönnig [18] described *S. alata* and *S. africana* based on only female specimens in South Africa. Both species are differentiated from the new species by distinctly smaller body size of female (4.61‒5.80 mm long in *S. alata* and *S. africana vs* 9.92‒12.1 mm long in *S. longicaudatum* n. sp.). In addition, *S. africana* differs from *S. longicaudatum* n. sp. by having the caudal alae of the female ending very near the tail tip (*vs* caudal alae ending at about half of tail length in *S. longicaudatum*). *Skrjabinema alata* differs from the new species by having a relatively longer oesophagus (oesophagus representing 12.8‒14.3 % of body length in *S. alata vs* representing 6.98‒8.78 % of body length in *S. longicaudatum*). Moreover, *S. longicaudatum* n. sp. can be easily distinguished from all its congeners by the unusually long tail in the female (tail 2.63‒3.13 mm, representing 23.0‒27.7 % of body length *vs* not over 1.60 mm, representing 6.68‒20.6 % of body length in the other species of *Skrjabinema*).

It is difficult to identify and discriminate the pinworms using traditional methods due to their extraordinary morphological similarity and sometimes the male worms being unavailable [26]. Molecular approaches have been employed for identification and discrimination of pinworms in some previous studies [24, 26‒32]. However, to date, genetic data of pinworms available in the GenBank database remain limited, which has hindered the further studies of DNA-based taxonomy, population genetics and phylogenetics of this group of nematode parasites.

In the present study, we amplified and sequenced the partial *18S*, *28S*, ITS and *cox*1 alignments of our specimens for future use in the molecular identification of this new species. There was no intraspecific nucleotide variation between different individuals of *S. longicaudatum* n. sp. in the partial *18S*, *28S*, ITS and *cox*1 sequences. However, a high level of nucleotide divergence was revealed between the new species and its congeners in *28S* (8.36%) and ITS (20.3–23.7%) regions, respectively. The more slowly evolving *18S* gene may be not suitable for species identification of *Skrjabinema*, because of very low level of interspecific nucleotide variation detected between different species of *Skrjabinema* (0.29–1.18%). However, the *18S* gene could be chosen to provide resolution at higher taxonomic levels. It is the first time to report the *cox*1 sequence of *Skrjabinema* species.

The systematic position of *Skrjabinema* is still under debate. Skrjabin [22] placed this genus into the subfamily Syphaciinae Railliet, 1916 in Syphaciidae Skrjabin & Schikhobalova, 1951. Erkulov & Moldopiyazova [33] proposed a new subfamily Skrjabinemiinae for the genera *Skrjabinema* and *Citellina*. Hugot [34] reduced the family Syphaciidae to a subfamily in Oxyuridae and did not recognise the validity of Skrjabinemiinae. The present phylogenetic analyses based on the partial *28S* and *18S*+*28S*+*cox*1 sequence data supported the genus *Skrjabinema* to be a member of the subfamily Oxyurinae, with a sister relationship with the genus *Oxyuris*, which agrees well with recent molecular phylogenetic results [26].

Our present knowledge of the composition of the nematode parasites of the Tibetan antelope remains limited. In the light of available literature, only three species of nematodes have been recorded from the Tibetan antelope, including *Nematodirus* sp., *Marshallagia mongolica* Schumakoviech, 1938 and *M. marshalli* (Ransom 1907) (Rhabditida: Strongyloidea) [9–11]. *Skrjabinema longicaudatum* n. sp. represents the first species of Oxyurida (pinworm) and the fourth nematode species reported from the Tibetan antelope.

## Conclusions

A new species of pinworm *Skrjabinema longicaudatum* n. sp. (Oxyurida: Oxyuridae) is described using light and scanning electron microscopy, based on specimens collected from the endangered Tibetan antelope. *Skrjabinema longicaudatum* n. sp. represents the first species of Oxyurida (pinworm) and the fourth nematode species reported from the Tibetan antelope. The nuclear and mitochondrial DNA sequences (i.e. 18S, 28S, ITS and *cox*1) were amplified and sequenced for molecular identification of this new species. Phylogenetic analyses using maximum likelihood (ML) inference based on 28S and 18S + 28S + *cox*1 sequence data suggested that the genus *Skrjabinema* should belong to the subfamily Oxyurinae (Oxyuroidea: Oxyuridae), instead of the subfamily Syphaciidae or Skrjabinemiinae in the traditional classification, as it formed a sister relationship to the genus *Oxyuris*. Our results contribute to the knowledge of the species diversity of parasites from the Tibetan antelope, provided useful genetic data for molecular identification and phylogeny of the Oxyuridae, and also clarified the systematic position of the genus *Skrjabinema*.

## Data Availability

The nuclear and mitochondrial DNA sequences of *Skrjabinema longicaudatum* n. sp. obtained in this study were deposited in the GenBank database under the accession numbers MW021552-MW021554 (*cox*1 sequences), MW020057-MW020059 (ITS sequences), MW020098-MW020100 (*28S* sequences), MW020179-MW020181 (*18S* sequences). Type specimens of the new species were deposited in College of Life Sciences, Hebei Normal University, Hebei Province under the accession numbers HBNU-N-2020M001-3L, and the Key Laboratory of Adaptation and Evolution of Plateau Biota, Northwest Institute of Plateau Biology, Chinese Academy of Sciences, Qinghai Province, under the accession numbers KLAEPB No.019001, China.

## Abbreviations

AEPB: Key laboratory of adaptation and evolution of plateau biota
SEM: Scanning electron microscopy
K2: Kimura 2-parameter
HKY: Hasegawa-Kishino-Yano
PCR: Polymerase chain reaction
ML: Maximum likelihood
*18S*: Small subunit ribosomal DNA
*28S*: Large subunit ribosomal DNA
ITS: Internal transcribed spacer
*cox*1: Cytochrome *c* oxidase subunit 1
L: Lip
i: Interlabia
tl: Triangular lateral lobes of lip
cp: Cephalic papillae
